# Psychiatric Symptoms and Fatigue in COVID-19 Survivors

**DOI:** 10.7759/cureus.45651

**Published:** 2023-09-20

**Authors:** Pravin Naphade, Pratistha Singh, Prajwal Rao, Shalesh Rohatgi, Suprakash Chaudhury, Sudhir Jadhav, Satish Nirhale

**Affiliations:** 1 Neurology, Dr. D. Y. Patil Medical College, Hospital & Research Centre, Pune, IND; 2 Psychiatry, Dr. D. Y. Patil Medical College, Hospital & Research Centre, Pune, IND; 3 Preventive Medicine, Dr. D. Y. Patil Medical College, Hospital & Research Centre, Pune, IND

**Keywords:** mhi, mental health and pandemic, modified fatigue impact scale, mental health after covid-19, mental health inventory, fatigue in covid-19, psychiatric symptoms in covid-19

## Abstract

Introduction: Psychiatric symptoms and fatigue are common after the coronavirus disease 2019 (COVID-19) illness. The cause of these symptoms is direct neuronal injury and indirect injury with immune-mediated inflammation. In addition, social factors also affect mental health.

Objective: We aim to compare psychiatric symptoms and fatigue between COVID-19 survivors and healthy controls.

Material and methods: We prospectively evaluated 100 COVID-19 survivors for anxiety, depression, positive affect, and behavior control using the Mental Health Inventory (MHI). Fatigue is assessed using the Modified Fatigue Impact Scale (MFIS) score. We compared them with 100 healthy controls.

Results: There was a significant statistical difference between the MHI score and individual components of MHI. Overall, MHI scores in cases and controls were 79.41 and 93.31, respectively, with a P value of less than 0.0001. Computed scores for anxiety, depression, behavior control, and positive affect of COVID-19 survivors showed statistically significant differences as compared to healthy controls. There was a weak association between hospital stay duration and poor MHI scores. Fatigue was significantly worse in COVID-19 survivors, with a mean score of 6.93 in cases and 5.35 in controls, with a P value of 0.0001. This was a cross-sectional study evaluating psychiatric symptom scores, but not establishing the diagnosis. It is suggested that appropriate treatment and counseling for these symptoms should be done.

Conclusions: Psychiatric symptoms and fatigue were significantly more common in COVID-19 patients after recovery from acute illness. It is a major contributing cause of morbidity other than organic complications of COVID-19 and requires attention in management.

## Introduction

Coronavirus disease 2019 (COVID-19) is an infectious disease caused by the SARS-CoV-2 virus, a positive-sense, single-stranded RNA virus. It can cause various symptoms ranging from common colds to severe acute respiratory syndrome (SARS) [1]. Severe acute respiratory syndrome (SARS) and Middle East respiratory syndrome (MERS) are also caused by coronaviruses and have been associated with neuropsychiatric manifestations such as delirium, depression, anxiety, and insomnia [2]. Coronaviruses have been shown to have the potential to cause direct neuronal injury as well as indirect injury with immune-mediated inflammation [3]. In addition, social factors such as isolation, stigma, and fear of illness can also affect mental health [4-6]. The evaluation of the direct neuropsychiatric complications and the indirect effects on mental health is needed for better treatment, mental healthcare planning, and prevention during potential subsequent pandemics [7]. In review, sub-syndromic mental health problems are a common response to the COVID-19 pandemic and need research in other affected countries [8]. Fatigue is a common symptom after an acute viral illness and can affect the quality of life. There are generally no objective markers to assess fatigue, and it has also been observed in a high percentage of COVID-19 survivors. In this case-control study, we aim to compare psychiatric symptoms and fatigue in post-discharge follow-up COVID-19 outpatients who recovered from acute illness with healthy controls.

## Materials and methods

Participants

Assuming a moderate effect size of 0.3 of the difference in the Mental Health Inventory (MHI) score between cases and controls at a significance level of 5% and power of 80%, the sample size was worked out to 82, which was rounded to 100. These 100 patients who survived COVID-19 (had positive reverse transcription-polymerase chain reaction (RT-PCR) test for COVID-19) in the last six months visited or were referred by other departments to the post-COVID-19 outpatient department (OPD). We also screened 100 healthy controls without a history of COVID-19. Controls were assumed to be free of COVID-19 infection. We excluded those with a history of fever, cough, breathlessness, or hospital admission in the past six months for similar complaints. RT-PCR test for COVID-19 was not done in controls. Both cases and controls were screened at post-COVID-19 OPD. Written consent was obtained. The study was approved by the Ethics Committee of Dr. D. Y. Patil Vidyapeeth, Pune (approval number: DYPV/EC/609/2020).

We evaluated the patients for psychiatric symptoms and the duration of hospital stay. For psychiatric symptoms, we used four components of the Mental Health Inventory (MHI): anxiety, depression, behavior control, and positive affect. The Mental Health Inventory developed by Veit and Ware [9] has 18 items to assess the overall mental health status. All 18 items are scored on a Likert scale. For each item, there are six responses scored as follows: 1, all of the time; 2, most of the time; 3, a good bit of the time; 4, some of the time; 5, a little of the time; and 6, none of the time. The overall reliability of the 18-item MHI scale is 0.93. The MHI generates one total score. The subscale and total scores range from 0 to 100, with higher scores indicating better mental health. We calculated overall and subgroup raw and computed MHI scores.

We also evaluated fatigue using the Modified Fatigue Impact Scale (MFIS). It is validated in many diseases [10,11]. Patients were asked how often fatigue has affected them during the past four weeks. Scoring was done on a Likert scale. The scale ascends from “never” to “rarely,” to “sometimes,” to “often,” to “almost always,” each scored 0-4, respectively. Five statements were offered. The sum provides a total score from 0 to 20. The higher the score, the worse the fatigue.

Statistical analysis

Statistical analysis was done to compare depression, anxiety, behavior control, and positive affect between cases and control, as well as to analyze fatigue severity. We tried to find out the correlation between the duration of hospital stay and psychiatric symptoms. Data were entered in Microsoft Excel 2019 (Microsoft Corporation, Redmond, WA, USA) and analyzed using MedCalc version 18.2.1 (MedCalc Software, Ostend, Belgium). Nominal variables were summarized by means of proportions and interval scale variables by mean (standard deviation (SD)) if normally distributed and median (interquartile range (IQR)) if not normally distributed. Normality was assessed using the Shapiro-Wilk test. In the case of interval scale data, if it was normally distributed, then a t-test was used (Welch modification was used if variances were not equal) and the Mann-Whitney U test if it was not normally distributed.

## Results

The study results are presented in Table 1.

**Table 1:** Raw MHI; computed MHI; computed anxiety, depression, behavior control, and positive affect; and MFIS scores of COVID-19 cases and healthy controls *Welch test (assuming unequal variances), **Mann-Whitney U test MHI: Mental Health Inventory, MFIS: Modified Fatigue Impact Scale, COVID-19: coronavirus disease 2019, SD: standard deviation, IQR: interquartile range

Scores	Cases (n=100) (mean (SD)/median (IQR))	Controls (n=100) (mean (SD)/median (IQR))	t/Mann-Whitney U test	P value
Raw MHI	79.41 (10.15)	93.31 (7.04)	11.25*	<0.0001
Computed MHI	68.19 (11.28)	83.68 (7.82)	11.29*	<0.0001
Computed anxiety	28 (20-40)	92 (84-96)	151**	<0.0001
Computed depression	72 (44-88)	92 (84-96)	2116.5**	<0.0001
Computed behavior control	65.9 (17.12)	84 (10.10)	9.11*	<0.0001
Computed positive affect	62.95 (22.51)	82.65 (11.13)	7.85*	<0.0001
MFIS	6.93 (2.49)	5.35 (2.58)	4.41	<0.0001

It demonstrates a significant difference in psychiatric symptom score and Modified Fatigue Impact Scale between cases and controls. In terms of demographic characteristics, as seen in Table 2, the mean age of cases was 34 years, which was similar to the mean age of controls of 34.5 years. Furthermore, 54% of cases were male and 46% were female, while in the controls, 47% were male and 53% were female. Although there were more males in the case group, this difference was not statistically significant (chi-square: P=0.32).

**Table 2:** Demographic characteristics of COVID-19 cases and healthy controls COVID-19: coronavirus disease 2019, IQR: interquartile range, CI: confidence interval

	Cases (n=100)	Controls (n=100)
Age (median (IQR))	34 (26-59)	34.5 (26-44)
Gender (number (%)) (95%CI)		
Males	54 (54%) (43.74%-64.02%)	47 (47%) (36.94%-57.24%)
Females	46 (46%) (35.98%-56.26%)	53 (53%) (42.76%-63.06%)

**Figure 1:**
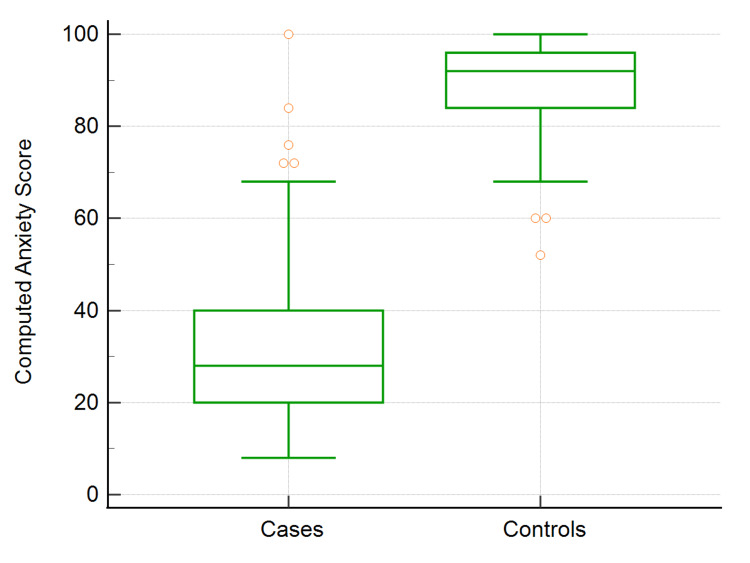
Computed anxiety scores of COVID-19 cases and healthy controls COVID-19: coronavirus disease 2019

As seen in Figure 1, the computed anxiety score for cases was 28, and for controls, it was 92, indicating a significant difference in the anxiety score.

**Figure 2:**
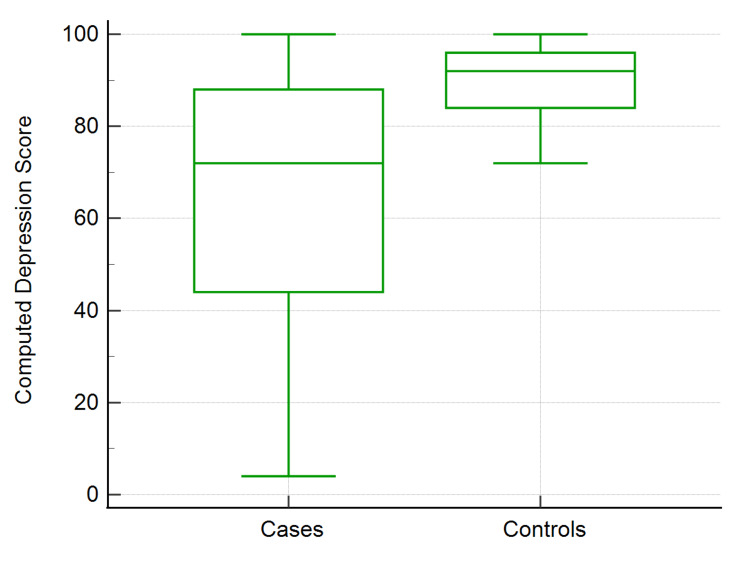
Computed depression scores of COVID-19 cases and healthy controls COVID-19: coronavirus disease 2019

Figure 2 shows that the computed depression score for cases was 72, and for controls, it was 92, showing a significant difference.

**Figure 3:**
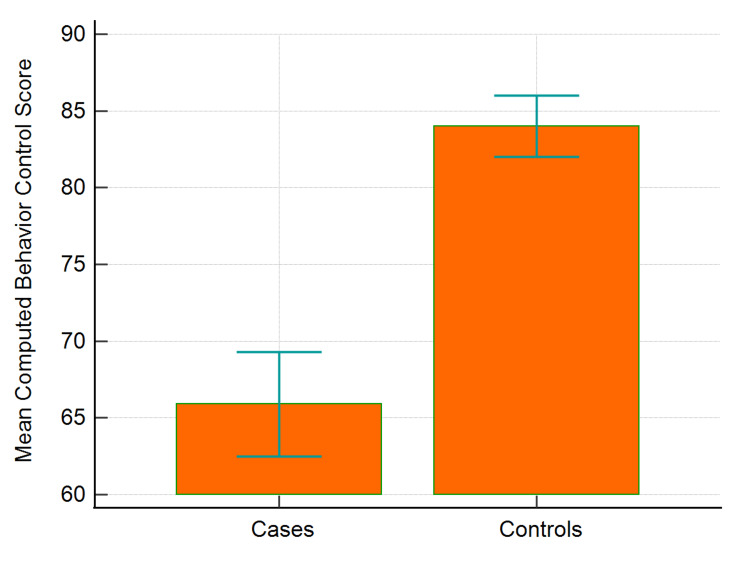
Mean computed behavior control scores of COVID-19 cases and healthy controls COVID-19: coronavirus disease 2019

Figure 3 shows that the mean computed behavior control scores for cases and controls were 65.9 and 84, respectively, showing a significant difference.

**Figure 4:**
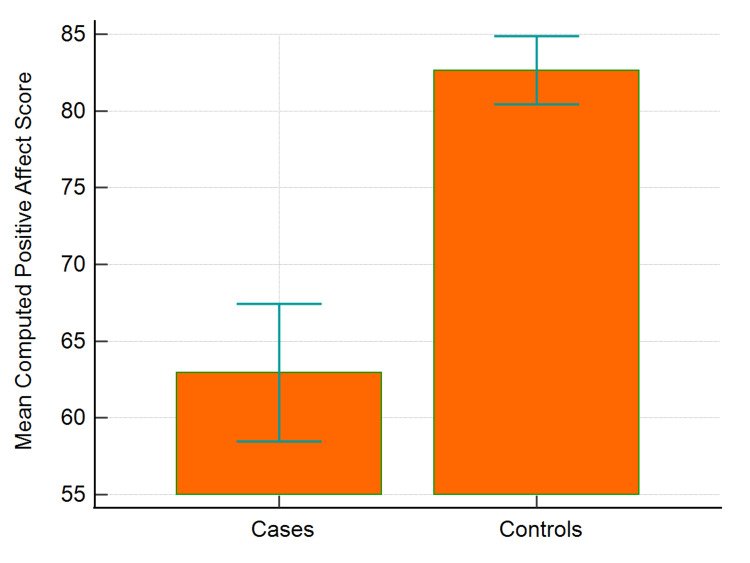
Mean computed positive affect scores of COVID-19 cases and healthy controls COVID-19: coronavirus disease 2019

Also, as seen in Figure 4, the mean computed positive affect scores for cases and controls were 62.95 and 82.65, respectively, demonstrating a significant difference.

The Modified Fatigue Impact Scale score in cases was 6.93, and in controls, it was 5.35; it shows a significant difference.

**Figure 5:**
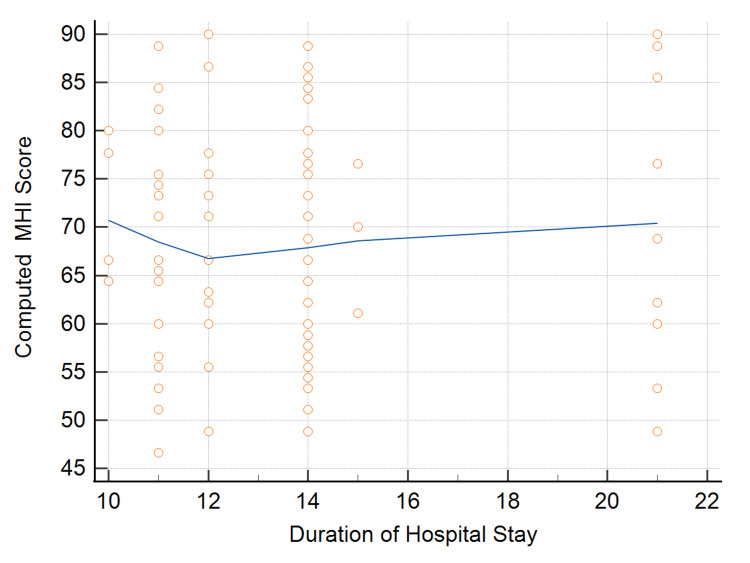
Association of hospital stay duration of COVID-19 cases and computed MHI score COVID-19: coronavirus disease 2019, MHI: Mental Health Inventory

The median duration of hospital stay among cases was 14 days, with an IQR of 11.5-14 days. The association of hospital stays with mean computed MHI is shown in Figure 5. Spearman’s rho was 0.0146 (P=0.89), showing a very weak positive association.

## Discussion

During the COVID-19 outbreak, psychiatric symptoms were commonly reported, and even in the post-illness period, these symptoms remained prevalent.

In our study, which is only a case-control study, we compared the symptoms in COVID-19 survivors on follow-up up to six months and healthy people who experienced the same time period of the COVID-19 pandemic. We found that there were significantly worse psychiatric symptoms in COVID-19 survivors on the overall MHI score (79.41 versus 93.31, P<0.0001) and also on an individual component of MHI compared to healthy controls. Computed anxiety score (28 versus 92, P<0.0001), computed depression score (72 versus 92, P<0.0001), computed behavior control score (65 versus 84, P<0.0001), and computed positive affect score (62.95 versus 82.65, P<0.0001) had statistically significant difference. Previous studies have shown a significant prevalence of psychiatric manifestations in post-COVID-19 patients. One study found psychiatric morbidities ranging from 10% to 35% in the post-illness stage [2]. High rates of anxiety (42%), depression (31%), and post-traumatic stress disorder (PTSD) (28%) have been reported in people who recovered after hospital admission for COVID-19 [12]. This was a situation after recovery from acute illness in an OPD follow-up.

Psychiatric symptoms can be caused by two mechanisms: immune-mediated and psychological stressors. The immune response to coronaviruses induces local and systemic production of cytokines, chemokines, and other inflammatory mediators [13]. COVID-19, severe acute respiratory syndrome (SARS), and Middle East respiratory syndrome (MERS) patients show activation of T-helper-1 cell function. In addition, COVID-19 patients also show elevated levels of T-helper-2 cell-secreted cytokines [14,15]. A study suggests a possible pathogenic role for monokine induced by gamma interferon (IFN-γ) (MIG) in long COVID [16].

Cytokine dysregulation has been studied in psychiatric disorders. Elevated levels of peripheral pro-inflammatory mediators such as interleukins and C-reactive proteins (CRPs) have been reported in bipolar disorder, as well as in other mood disorders [17]. The relationship between inflammatory profile and the hypothalamic-pituitary-adrenal (HPA) axis has been explored. Cytokines can cross the blood-brain barrier (BBB) and act on various areas of the central nervous system (CNS), including this axis. HPA axis dysfunction with elevated levels of pro-inflammatory cytokines can affect neuronal plasticity with a negative impact on mood symptoms and cognition [18].

Psychological stressors such as social isolation, fear of illness, stigma, and uncertainty can also cause psychiatric symptoms [19]. A study in a community in Spain detected higher levels of symptoms on the Depression, Anxiety, and Stress Scale (DASS) after the stay-at-home order was issued, and these symptoms were predicted to increase as confinement continued [20].

Fatigue is also a common symptom during the post-COVID-19 period. In this study, we found significantly worse fatigue scores in COVID-19 survivors than in healthy controls (6.93 versus 5.35, P=0.0001). In another study, a similar finding was seen in patients treated in the hospital for severe COVID-19 who experienced disabling fatigue, with a mean rating of 4.8 out of 10. A high rate of illness‐related fatigue was reported, with 72% of participants in the ICU group and 60.3% in the ward group [20,21]. Elevated levels of cytokines might be contributing to fatigue. It was found that treatment with pro-inflammatory interferon-α induces fatigue in cancer patients [22], and treatment with the anti-tumor necrosis factor (TNF)-α drug etanercept reduces fatigue in patients with psoriasis [23]. Psychiatric symptoms and fatigue were common during the acute illness phase and continued afterward.

We found no difference in MHI scores in male and female patients. Also, there is no correlation between hospital stay and the MHI total score. A past study found an inverse correlation between the duration of hospital stay, anxiety, depression, and post-traumatic stress disorder (PTSD) [12].

The limitation of our study is that it is a cross-sectional study, and these scores provide objectivity for psychiatric symptoms, but they do not establish a diagnosis of psychiatric illness.

## Conclusions

The COVID-19 pandemic was a stressful period. Psychiatric symptoms and fatigue were significantly more common in COVID-19 patients after recovery from acute illness. In our study, the mean total Mental Health Inventory (MHI) score, mean subcomponent MHI score, and Modified Fatigue Impact Scale score were significantly higher in COVID-19 patients as compared to healthy controls. The causes for this affection were organic, such as the virus itself involving the nervous system and inflammatory response to the virus. Also, social factors such as isolation, fear of illness, and loss of jobs contributed to psychiatric symptoms and fatigue. The treatment of COVID-19 patients requires managing organic manifestations in the acute stage and attention to psychiatric symptoms and fatigue after recovery from the acute stage of illness.
